# Dabigatran Suppresses PAR-1/SphK/S1P Activation of Astrocytes in Experimental Autoimmune Encephalomyelitis Model

**DOI:** 10.3389/fnmol.2020.00114

**Published:** 2020-06-30

**Authors:** Rong Chen, Xing Cao, Wenxiu Luo, Haodi Yang, Xinya Luo, Juming Yu, Jiaming Luo

**Affiliations:** ^1^Department of Microbiology and Immunology, North Sichuan Medical College, Nanchong, China; ^2^Department of Neurology, Affiliated Hospital of North Sichuan Medical College, Nanchong, China; ^3^Department of Anesthesia, North Sichuan Medical College, Nanchong, China; ^4^School of Psychiatry, North Sichuan Medical College, Nanchong, China

**Keywords:** multiple sclerosis, experimental autoimmune encephalomyelitis, dabigatran, protease-activated receptors, sphingosine-1-phosphate, coagulation

## Abstract

Multiple sclerosis (MS) is an inflammatory autoimmune disease affecting the central nervous system (CNS) that currently does not have any effective treatment. Experimental autoimmune encephalomyelitis (EAE) is often employed as a model to mimic the clinical manifestations of MS, mainly CNS demyelination. Coagulation is known to participate in crosstalk with inflammation and autoimmunity. We herein explored the correlation between the coagulation cascade and CNS immune diseases *in vitro* using primary astrocytes isolated from mice and *in vivo* using a mouse model of EAE. We showed that dabigatran, a clinical oral anti-coagulant drug, suppressed the thrombin-induced activation of astrocytes, and the underlying mechanisms are related to the activity of protease-activated receptor-1 (PAR-1), sphingosine-1-phosphate (S1P), and sphingosine kinases (SphKs). Importantly, dabigatran effectively recovered neurological function, reduced inflammation in the spinal cord, and prevented spinal cord demyelination caused by EAE. We suggest that dabigatran, a specific inhibitor of thrombin, antagonized the effect of thrombin in astrocytes by limiting the activation of PAR-1, in turn downregulating SphK1 and disrupting S1P receptor signaling. These findings reveal critical information about the relationship between coagulation mechanisms and CNS immune diseases and will contribute to the clinical translation and development of therapeutic strategies against MS.

## Introduction

Multiple sclerosis (MS) is an inflammatory autoimmune disease of the central nervous system (CNS) mostly occurring in young and middle-aged people aged 20–40 years (Ransohoff et al., 2015). It exhibits a chronic pathological course, with repeated recurrence affecting multiple lesions of the CNS, resulting in severe neurological defects. Researchers believe that MS occurs in genetically susceptible individuals under the influence of environmental factors and is mediated by autoimmune demyelination of the CNS, further causing gliosis, axonal injury, necrosis, and neuronal death (Dendrou et al., 2015). At present, the etiology of MS is unclear, and its pathogenesis is complex. Thus, drugs currently adopted in MS are only symptomatic, and treatment options are limited. Immunomodulation therapy has recently shown progress, but the therapeutic effect is far from ideal from clinical perspectives. To study the pathogenesis of MS, the experimental autoimmune encephalomyelitis (EAE) model has been developed to mimic the phenomenon of demyelination in the CNS (Rivers et al., 1933). In this model, autoimmunity-prone female C57BL6 mice are induced by myelin oligodendrocyte glycoprotein (MOG) to trigger autoimmune responses (Kataoka et al., 2005). This in turn causes pathological demyelination and activation of cytotoxic T lymphocytes to attack the myelin sheath of the CNS, simulating the clinical manifestation and symptoms of MS (Jagessar et al., 2015; Peschl et al., 2017).

Astrocytes are glial cells that, under normal circumstances, support the CNS by maintaining metabolic balance and the integrity of the blood-brain barrier (Turner and Adamson, 2011). In pathological conditions such as CNS injury, astrocytes are activated and express a large amount of glial fibrillary acid protein (GFAP). Astrocyte activation is a critical factor in maintaining immune inflammation in the CNS, and recent studies have linked astrocytes to the pathogenesis of MS and EAE (Itoh et al., 2018; Ponath et al., 2018; Brambilla, 2019). After oligodendrocyte myelin sheath destruction, oxidative stress, and the release of free radicals further stimulate the microglia to release interleukin (IL)-1β and other inflammatory cytokines, in turn activating an abundance of astrocytes (Cekanaviciute and Buckwalter, 2016). The activated astrocytes function to amplify and maintain inflammation and are the main effector cells of inflammatory attack (Xie and Yang, 2015).

Large-scale proteomic analysis has revealed that the pathogenesis of MS is associated with the dysregulation of proteins in the coagulation cascade (Han et al., 2008). The thrombin inhibitor hirudin and the anti-coagulant warfarin are effective in reducing demyelinating lesions and alleviating EAE symptoms in experimental mice by inhibiting the release of inflammatory cytokines in astrocytes (Han et al., 2008; Stolz et al., 2017). Protease-activated receptors (PARs) are important components of the blood coagulation cascade whose functions are interwoven with the inflammatory response (Asehnoune and Moine, 2013; Foley and Conway, 2016). Specifically, it was reported that the inhibition of PAR-1 effectively alleviated the symptoms of EAE by stabilizing the blood-brain barrier (Kim et al., 2015). Also, the activation of PAR-1 was directly correlated with the activity of sphingosine-1-phosphate (S1P; Tauseef et al., 2008), the formation of which is catalyzed by sphingosine kinases (SphK1 and SphK2; Maceyka et al., 2012). Studies have found that the S1P signaling pathway is involved in the pathological process of autoimmune diseases, tumors, vascular diseases, and other conditions (Proia and Hla, 2015). In the CNS, S1P signaling is closely related to diseases such as ischemic brain injury, glioma, and MS. Microglia activation is reportedly associated with increased S1P content after spinal cord injury (Kimura et al., 2007), and the expression of SphK1 was shown to increase in MS lesions of lipopolysaccharide (LPS)-induced rat astrocytes (Fischer et al., 2011), suggesting that increased S1P production may be a factor that leads to astrogliosis in MS.

Dabigatran is a commercial anti-coagulant that functions as a competitive and reversible inhibitor of thrombin. It is formulated as a prodrug (dabigatran etexilate) and is converted into the active form after oral administration (Supplementary Figure S1). We hypothesized that anti-coagulants can ameliorate CNS inflammation by inhibiting the PAR-1 receptor, in turn downregulating the gene and protein expression of SphK1 and S1P to suppress the proliferation and activation of astrocytes. Based on this hypothesis, we compared the effect of dabigatran and SCH 530348, an anti-coagulating agent that functions by antagonizing PAR-1, in regulating astrocyte activation and inflammatory response through the S1P/SphK axis *in vitro* and *in vivo*, to elucidate the correlation between coagulation mechanisms and CNS immune diseases.

## Materials and Methods

### Isolation and Primary Culture of Astrocytes

The fetus was removed from pregnant specific-pathogen-free C57BL6 mice (provided by the Hubei Provincial Center for Disease Control and Prevention) at 20 days of gestation. The brain was extracted from the fetus, and the soft meninges and blood vessels were removed using curved tweezers under a stereomicroscope. The cerebral cortex was gently transferred to an Eppendorf tube containing Dulbecco’s modified Eagle medium (DMEM)/F12 medium (SH30023.01, Hyclone). Brain tissues were cut into small pieces and mixed with the same volume of 0.25% trypsin (PAB180002, Bioswamp, Wuhan, China) and 0.2% collagenase (P2636, Sigma-Aldrich, St. Louis, MO, USA). After 30 min of incubation at 37°C, 5 ml of DMEM/F12 was added and thoroughly mixed with the tissue. The mixture was centrifuged at 1,000 rpm for 5 min, the supernatant was discarded, and the cells were resuspended in DMEM/F12 medium. The cell suspension was passed through a 200-mesh stainless steel wire screen and the previous step was repeated twice. The isolated cells were cultured in DMEM/F12 medium containing 10% fetal bovine serum (10270-106, Gibco) and incubated for 90 min in 5% CO_2_. Fibroblasts were removed by differential adherence separation, and the remaining cells were resuspended at 5 × 10^5^ cells/ml. Cells were subjected to astrocyte purification after 12 days of culture by shaking at 250 rpm at 37°C for 12 h. The medium containing detached cells was removed to obtain purified adherent astrocytes.

### *In vitro* Cell Treatment

Purified astrocytes were subjected to treatment with either LPS or thrombin to induce activation. Astrocytes at 70% confluence were treated with LPS (L2880, Sigma-Aldrich) at 100 ng/ml or human thrombin (T9326, Sigma-Aldrich) at 1 U/ml. For anti-coagulant treatment, LPS- or thrombin-induced cells were simultaneously incubated with dabigatran etexilate (Dab; D126683, Aladdin, Shanghai, China) at 500 nmol/L. As a positive control, LPS-induced astrocytes were treated simultaneously with SCH 530348 (S8067, Selleck Chemicals, Houston, TX, USA), an inhibitor of PAR-1 (hereafter denoted as PAR-1-inh), at 1,000 nmol/L. All cell treatment occurred for 1 or 6 h at 37°C in an atmosphere containing 5% CO_2_.

### Immunofluorescence

At 0, 1, and 6 h after treatment, astrocytes were visualized for GFAP and proteins associated with S1P *via* immunofluorescence staining. Cells seeded on coverslips were washed twice with phosphate-buffered saline (PBS) and fixed in 4% paraformaldehyde for 30 min, after which they were washed three times with PBS for 3 min each. Permeabilization was performed for 20 min using 0.5% Triton X-100 in PBS and the cells were washed three times with PBS for 3 min each. The cells were then blocked with 5% bovine serum albumin at 37°C for 1 h and incubated overnight at 4°C with primary antibodies against GFAP (mouse, 1:100, ab10062, Abcam, Cambridge, UK), PAR-1 (mouse, 1:100, NB1-71770-SS, Novus Biologicals, Centennial, CO, USA), S1P receptor 1 (S1PR1, rabbit, 1:150, NB120-11424, Novus Biologicals), SphK1 (rabbit, 1:50, ab71700, Abcam), and SphK2 (rabbit, 1:100, 1-SP030-02, Quartett, Berlin, Germany). Thereafter, the cells were washed three times with PBS for 5 min each and incubated at 37°C for 1 h with AlexaFluor 594-conjugated goat anti-mouse (PAB160019, Bioswamp) or AlexaFluor 488-conjugated Affinipure goat anti-rabbit (PAB160027, Bioswamp) or goat anti-mouse (PAB160017, Bioswamp) secondary antibodies. The cells were then washed five times with PBS for 3 min each and stained with 20 μl of 4′,6-diamidino-2-phenylindole solution (PAB180018, Bioswamp). The coverslips were mounted on microscope slides and visualized using a fluorescence microscope. Positive fluorescence was quantified as the relative mean integrated optical density (IOD) using ImagePro Plus software.

### Quantitative Reverse Transcription Polymerase Chain Reaction (qRT-PCR)

RNA was extracted using TRIzol (15596026, Ambion Inc., Foster City, CA, USA) and reverse-transcribed into cDNA using the RevertAid First Strand cDNA Synthesis Kit (K1622, Thermo Scientific). qRT-PCR was performed using the SYBR Green PCR kit (KM4101, KAPA Biosystems, Wilmington, MA, USA) with the following primer sequences: S1P forward, 5′-CGCAAGAACATCTCCAA-3′ and reverse, 5′-GCAGCCCACATCTAACA-3′; IL-1β forward, 5′-CTTCAGGCAGGCAGTA-3′ and reverse, 5′-ATCCCATGAGTCACAGAG-3′; PAR-1 forward, 5′-CGGACAGAGTTGATGGTG-3′ and reverse, 5′-AAGGAGCAGATAGGTAGCC-3′; S1PR1 forward, 5′-CGCAAGAACATCTCCA-3′ and reverse, 5′-GCAGCCCACATCTAACA-3′; SphK1 forward, 5′-GGCTGCGGCTCTATTCT-3′ and reverse, 5′-GGTGCCCACTGTGAAAC-3′; SphK2 forward, 5′-CTTTACGAGGTGCTGAATGG-3′ and reverse, 5′-AGAAGAAGCGAGCAGTTGA-3′; GAPDH forward, 5′-CCTTCCGTGTTCCTAC-3′ and reverse, 5′-GACAACCTGGTCCTCA-3′. The experimental conditions were as follows: initial denaturation at 95°C for 3 min; 39 cycles of denaturation at 95°C for 5 s, annealing at 56°C for 10 s, and extension at 72°C for 25 s; and a final extension at 65°C for 5 s and 95°C for 50 s. Data analysis was carried out using the qbase plus software *via* the 2^−ΔΔCt^ method.

### Enzyme-Linked Immunosorbent Assay (ELISA)

ELISA was performed using assay kits for IL-1β (MU30369, Bioswamp) and S1P (MU30786, Bioswamp), and all reagents were provided in the kits. The wells of a well-plate were coated with antibodies against the protein of interest. Medium from cultured cells was collected and 40 μl of the medium was added to each well. After incubation, 10 μl of biotinylated antibodies against the protein interest were added to the wells, after which, 50 μl of horseradish peroxidase conjugate reagent was added to each well. The plate was incubated for 30 min at 37°C, and the chromogen was added to the wells. The plate was incubated for 10 min at 37°C and 50 μl of stop solution was added to stop the reaction. The optical density of the wells was measured at 450 nm and the concentration of the released proteins was calculated using a standard curve.

### Western Blot

Cell or tissue samples were lysed using radioimmunoprecipitation assay buffer. Protein content was quantified using a bicinchoninic acid assay kit (PAB180007, Bioswamp), and 20 μg of proteins were loaded onto a 12% gel for sodium dodecyl sulfate-polyacrylamide gel electrophoresis. The proteins were transferred to polyvinylidene fluoride membranes and blocked with 5% skim milk at room temperature for 2 h. Then, the membranes were incubated overnight at 4°C with primary antibodies against S1P (rabbit, 1:1,000, ab140592, Abcam), PAR-1 (mouse, 1:1,000, NB1-71779-SS, Novus Biologicals), S1PR1 (rabbit, 1:1,000, NB120-11424, Novus Biologicals), SphK1 (rabbit, 1:1000, ab71700, Abcam), SphK2 (rabbit, 1:1,000, 1-SP030-02, Quartett), IL-1β (rabbit, 1:500, ab200478, Abcam), and GAPDH (rabbit, 1:5,000, 10494-1-AP, Proteintech, Rosemont, IL, USA). The membranes were then washed three times with PBS/Tween 20 for 3 min each and incubated at room temperature for 1 h with goat anti-rabbit IgG (1:10,000, PAB150011, Bioswamp) or goat anti-mouse IgG (1:10,000, PAB150009, Bioswamp) secondary antibodies. After three washes in PBS/Tween 20 for 5 min each, the membranes were incubated with an enhanced chemiluminescence reagent (WBKLS0010, Millipore) in the absence of light and visualized using an automatic analyzer (Tanon-5200, Tanon, Shanghai, China).

### *In vivo* EAE Model

All animal experiments were approved by the institutional review board and performed following the Guidelines for Animal Care and Use of the Model Animal Research Institute at Wuhan Myhalic Biotechnology Co., Limited (approval no. HLK-20181015-01). The *in vivo* model of EAE was established in female C57BL6 mice (*n* = 6 per group) following the protocol described by Kataoka et al. (2005), with slight modifications. A solution of MOG_35–55_ polypeptide (sequence: MEVGWYRSPFSRVVHLYRNGK; M4939, obtained from Sigma-Aldrich) was prepared in PBS at 0.01 mol/L and emulsified in 200 μl of Freund’s Complete Adjuvant (F5881, Sigma-Aldrich) containing *Mycobacterium tuberculosis* H37Ra, to achieve a final concentration of 300 μg/ml. The solution was repeatedly mixed using a glass syringe until it was completely emulsified. Immunization was performed *via* a subcutaneous injection of the emulsified mixture at the limbs of the experimental mice at 0.2 ml per mouse. Control mice were injected with Freund’s Complete Adjuvant mixed with saline instead of MOG_35–55_. The mice were intraperitoneally injected with 500 ng of pertussis toxin immediately and 48 h after immunization. Ten days after immunization (defined as the onset of EAE), Dab (10 mg/g), or SCH 530348 (dissolved in dimethyl sulfoxide at 10 μg/kg) was administered orally daily, with physiological saline as vehicle control. Thirty days after immunization, the mice were anesthetized with 1% sodium pentobarbital and at a dose of 0.15 ml/10 g, after which the spinal cord was extracted for subsequent experiments.

### Assessment of Neurological Function

From the day of immunization, the mice were monitored for neurological behavior and given a clinical score based on the following criteria: 0, unaffected; 1, tail limpness; 2, failure on an attempt to roll over; 3, partial paralysis; 4, complete paralysis, and 5, moribund.

### Tissue Preparation

The spinal cord of experimental rats was fixed in 10% neutral formalin for 1 day and cut into pieces of approximately 1.5 cm × 1.5 cm × 0.3 cm. After paraffin embedding, the tissues were sectioned at 4 μm and mounted onto microscope slides. Before staining, the sections were deparaffinized and washed in water for 2 min.

### Hematoxylin and Eosin (H&E) Staining

H&E staining was performed to observe tissue morphology and inflammatory infiltration. Deparaffinized spinal cord tissue sections were immersed in hematoxylin solution (PAB180015, Bioswamp) for 5 min and washed in running water for 2 min to remove the residual dye. The sections were then placed in 1% hydrochloric alcohol for 3 s, washed briefly, placed in bluing solution for 10 s, and washed under running water for 30 s. Thereafter, the sections were stained with 0.5% eosin solution (PAB180016, Bioswamp) for 3 min. The sections were washed briefly with distilled water, 80% ethanol for 30 s, 95% ethanol for 30 s, and briefly in anhydrous ethanol and xylene, then mounted in neutral balsam gum. Stained tissues were observed using a microscope (MD1000, Leica, Wetzlar, Germany).

### Luxol Fast Blue (LFB) Staining

An LFB staining kit (Servicebio, Wuhan, China) was applied to stain myelin tissue and evaluate demyelination. Before staining, the LFB solution was preheated at 60°C for 30 min. Spinal cord tissue sections were then stained with LFB solution for 3 h at 60°C and cooled for 15 min. After the sections were washed in tap water for 2 min, they were differentiated *via* alternate immersion (approximately 2 s) in 70% ethanol and lithium carbonate solution (provided in the kit) at room temperature. The degree of differentiation was controlled using a microscope. After differentiation, the sections were washed with tap water and immersed three times in anhydrous ethanol for 5 min each and twice in xylene for 5 min each. The sections were sealed using neutral balsam gum and observed using a microscope (MD1000, Leica).

### Statistical Analysis

*In vitro* experiments were performed in triplicates (*n* = 3) and *in vivo* experiments were performed in six replicates (*n* = 6). The data are presented as the mean ± standard deviation (SD). Statistical analysis was carried out by one-way analysis of variance with Tukey’s test for multiple comparisons using OriginPro 8. *p* < 0.05 is considered statistically significant.

## Results

### Dabigatran Suppressed LPS- and Thrombin-Induced Inflammation and S1P Signaling in Astrocytes

**Figure 1:**
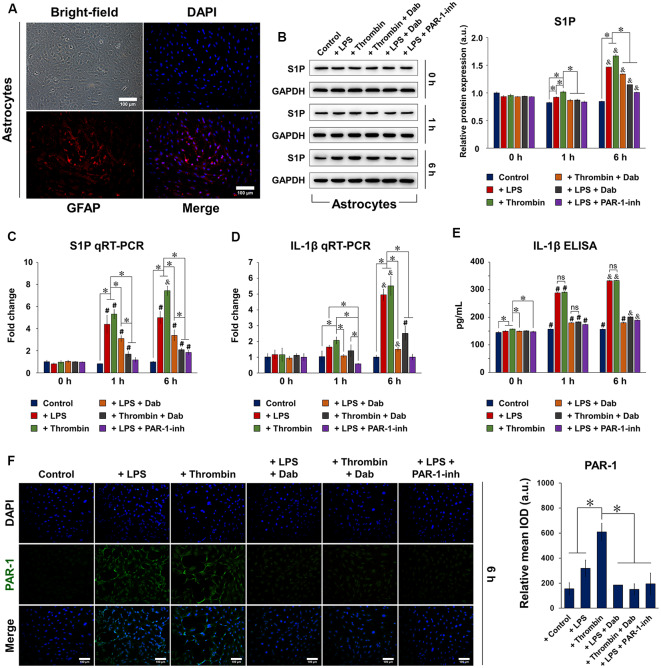
Expression of sphingosine-1-phosphate (S1P), interleukin (IL)-1β, and protease-activated receptor-1 (PAR-1) in isolated astrocytes. **(A)** Isolated and purified astrocytes were identified by bright-field microscopy and immunofluorescence for glial fibrillary acid protein (GFAP; red). Nuclei (DAPI) were labeled in blue. Scale bar = 100 μm.** (B)** Western blot and quantitative reverse transcription polymerase chain reaction (qRT-PCR) of the protein and mRNA expression, respectively, of S1P 0, 1, and 6 h after astrocytes were treated with lipopolysaccharide (LPS) or thrombin and Dabigatran (Dab) or PAR-1-inh (LPS only). **(D)** qRT-PCR and **(E)** enzyme-linked immunosorbent assay (ELISA) of the mRNA expression and secretion, respectively, of IL-1β 0, 1, and 6 h after astrocytes were treated with LPS or thrombin and Dab or PAR-1-inh (LPS only). **(F)** Immunofluorescence of astrocytes and quantification of relative mean integrated optical density (IOD; green fluorescence). PAR-1 was stained in green and nuclei (DAPI) were stained in blue. Scale bar = 100 μm. For **(B–F)**, data are presented as the mean ± SD (*n* = 3). *p < 0.05; ^#^*p* < 0.05 vs. the same group at 0 h; ^&^*p* < 0.05 vs. the same group at 1 h. ns, not significant.

Isolated and purified astrocytes were identified using bright-field and fluorescence microscopy (Figure 1A), showing an abundance of positive GFAP staining. We first examined the protein and gene expression of S1P (Figures 1B,C, respectively) in astrocytes at 0, 1, and 6 h after stimulation. Notably, the protein and gene expression of S1P were both significantly increased by 6 h with LPS or thrombin stimulation, but the use of Dab counteracted the effect of the stimulants by decreasing the protein and gene expression of S1P. We then evaluated the gene expression (Figure 1D) and secretion (Figure 1E) of IL-1β in astrocytes using qRT-PCR and ELISA, respectively, at 0, 1, and 6 h after stimulation. Astrocytes showed a significant increase in the gene expression and secretion of IL-1β after 1 h of stimulation with LPS or thrombin, and a further increase was observed at 6 h. Particularly in terms of IL-1β gene expression, a drastic increase was observed at 6 h compared to 1 h after LPS or thrombin stimulation. Upon Dab treatment in LPS- and thrombin-stimulated astrocytes, IL-1β gene expression and secretion were significantly downregulated. In the case of both S1P and IL-1β, comparison with the PAR-1-inh as a positive control showed that the effect of Dab was similar to that of PAR-1-inh at all time points. These results signify that LPS- and thrombin-induced inflammation and S1P activation in astrocytes were attenuated by Dab, which showed similar effects as those of PAR-1-inh.

**Figure 2:**
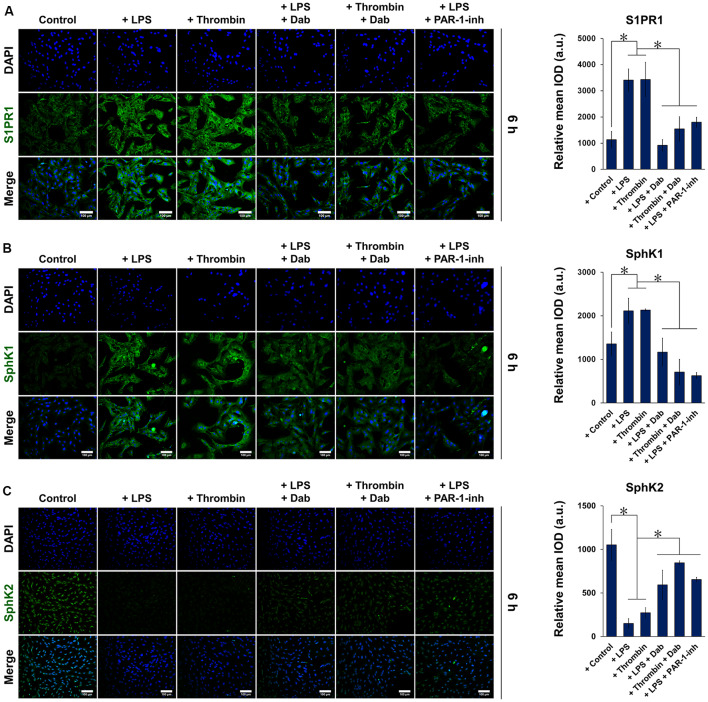
Immunofluorescence of proteins related to S1P signaling in astrocytes. Astrocytes were treated with LPS or thrombin, with or without Dab or PAR-1-inh (LPS only) for 6 h. Cells were stained for **(A)** S1P receptor 1 (S1PR1), **(B)** sphingosine kinase (SphK1), and **(C)** SphK2 in green and nuclei (DAPI) in blue. Scale bar = 100 μm. Green fluorescence was quantified by measuring the relative mean IOD. The data are presented as the mean ± SD (*n* = 3), *p < 0.05.

We then performed immunofluorescence staining of PAR-1 in astrocytes and observed that those treated with LPS or thrombin for 6 h showed positive expression of PAR-1, as indicated by the green fluorescence signal (Figure 1F; images of astrocytes treated with Dab or PAR-1-inh only are shown in Supplementary Figure S2). Simultaneous incubation with Dab for 6 h reduced the expression of PAR-1 in both LPS- and thrombin-treated astrocytes, and the effect was similar to that of PAR-1-inh. The expression of S1P signaling-related proteins was also examined by immunofluorescence (Figure 2). We noticed that LPS and thrombin significantly elevated the expression of S1PR1 (Figure 2A) and SphK1 (Figure 2B), and treatment with Dab or PAR-1-inh counteracted the effect of LPS and/or thrombin on astrocytes. However, SphK2 exhibited the opposite behavior as SphK1, showing decreased expression upon LPS or thrombin stimulation and upregulation with Dab or PAR-1-inh treatment (Figure 2C; immunofluorescence images for PAR-1, S1PR1, SphK1, and SphK2 at 0 h and 1 h of treatment are shown in Supplementary Figures S3–S6).

### Dabigatran Regulated the Expression of Components of PAR-1/SphK/S1P Axis

**Figure 3:**
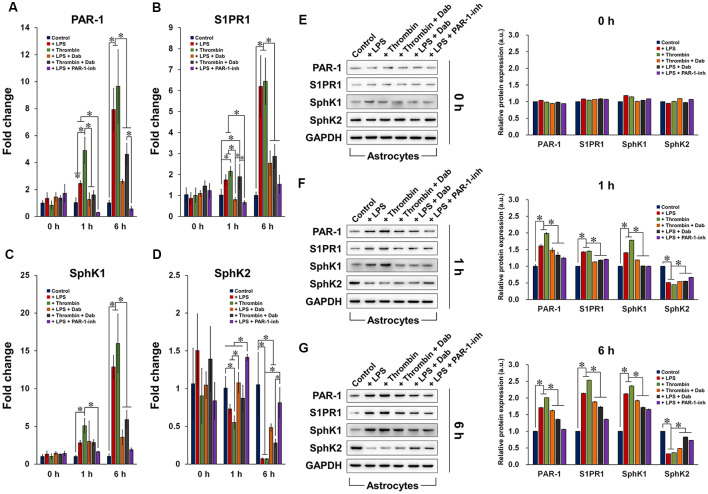
Gene and protein expression of components of the PAR-1/SphK/S1P axis. qRT-PCR analysis of the gene expression of **(A)** PAR-1, **(B)** S1PR1, **(C)** SphK1, and **(D)** SphK2 in astrocytes treated with LPS or thrombin, with or without Dab or PAR-1-inh (LPS only), for 0 h, 1 h, or 6 h. Western blot detection and quantification of the protein expression of PAR-1, S1PR1, SphK1, and SphK2 in astrocytes treated with LPS or thrombin, with or without Dab or PAR-1-inh (LPS only), for **(E)** 0 h, **(F)** 1 h, or **(G)** 6 h. The data are presented as the mean ± SD (*n* = 3), *p < 0.05.

Astrocytes induced by LPS or thrombin with or without anti-coagulant treatment were subjected to qRT-PCR (Figures 3A–D) and western blot (Figures 3E–G) to examine the gene and protein expression of PAR-1, S1PR1, SphK1, and SphK2. In general, the gene and protein expression of PAR-1, S1P, and SphK1 were upregulated by LPS and thrombin after 1 h of treatment and were further elevated by 6 h. SphK2 showed the opposite and was downregulated by LPS and thrombin over time, which was anticipated as SphK1 and SphK2 function in opposite manners (Maceyka et al., 2005). The administration of Dab and PAR-1-inh counteracted the effect of LPS and thrombin in terms of both gene and protein expression of the aforementioned factors, and the change was more evident at 6 h compared to that at 1 h of treatment. Particularly in terms of gene expression, PAR-1-inh, as a specific inhibitor of PAR-1, suppressed the gene expression of PAR-1 more strongly than did Dab at 1 h and 6 h. Otherwise, the effect of Dab was similar to that of PAR-1-inh in regulating the gene expression of S1PR1, SphK1, and SphK2. Together, these results signify that anti-coagulants can alter the expression of components in S1P/SphK-mediated signaling and that the underlying mechanisms are associated with the regulation of PAR-1.

### Dabigatran Improved Neurological Behavior and Suppressed Spinal Inflammation and Demyelination in EAE-Induced Mice

**Figure 4:**
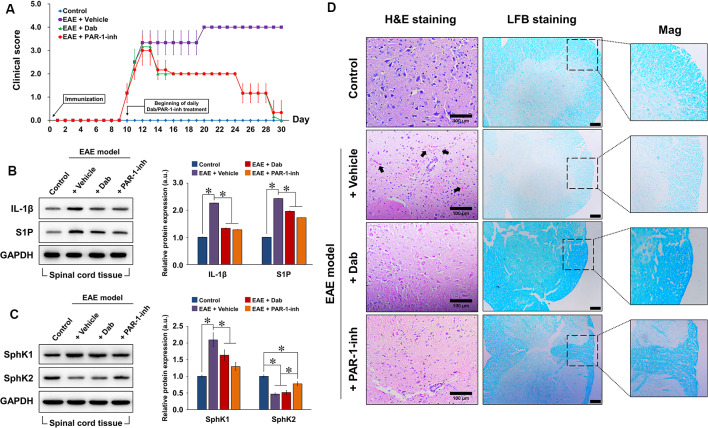
Construction of the *in vivo* experimental autoimmune encephalomyelitis (EAE) model. Experimental mice were induced by EAE and treated with physiological saline (vehicle), 10 mg/g Dab, or 10 μg/kg PAR-1-inh. **(A)** Clinical evaluation of neurological function was carried out daily for over 30 days. 0, unaffected; 1, tail limpness; 2, failure on attempt to roll over; 3, partial paralysis; 4, complete paralysis, and 5, moribund. Data are presented as the mean ± SD of six replicates. Western blot evaluation and quantification of the protein expression of **(B)** IL-1β, S1P, **(C)** SphK1, and SphK2 in spinal cord tissues after 30 days of EAE modeling, with or without treatment. **(D)** Hematoxylin and eosin (H&E) and luxol fast blue (LFB) staining of spinal cord tissue 30 days after EAE modeling, with or without treatment. Arrows point to pink areas indicative of inflammatory infiltration. The intensity of blue color in the LFB images represents the degree of demyelination (lighter = greater demyelination). Mag = magnification of specific areas of interest in LFB images. Scale bar = 100 μm for H&E and 50 μm for LFB. For **(B,C)**, the data are presented as the mean ± SD (*n* = 6), *p < 0.05.

After EAE was induced in the experimental mice, we monitored the neurological behavior of the animals daily based on their mobility and ability to perform basic tasks (Figure 4A). A clinical score was assigned from 0 to 5. No neurological defect was observed in the control mice (non-EAE) during the 30-day experimental period, whereas the clinical score gradually increased in EAE-induced mice from day 9 onward, indicating that neurological function was impaired. On day 10, the daily administration of physiological saline, Dab, or PAR-1-inh was initiated in EAE-induced mice. Vehicle-treated mice continuously showed increasing clinical scores. However, from day 13 onward, mice treated with Dab or PAR-1-inh exhibited a gradual decrease in clinical score, signifying that both anti-coagulants reversed the damaging effect of EAE on the neurological function to a certain degree.

Next, we assessed the protein expression of IL-1β, S1P, SphK1, and SphK2 in spinal cord tissues extracted from the experimental mice after 20 days of EAE induction (Figures 4B,C). Compared to the control, EAE-induced mice showed a remarkable increase in IL-1β, S1P, and SphK1 expression and a decrease in SphK2, implicating the activation of inflammatory and S1P-related signaling pathways. With the continuous administration of Dab or PAR-1-inh, IL-1β, S1P, and SphK1 expression were significantly suppressed while that of SphK2 was upregulated, confirming the influence of the anti-coagulants on inflammation and S1P signaling.

To assess the effect of anti-coagulation on inflammatory infiltration and demyelination, we performed H&E and LFB staining, respectively, on extracted spinal cord tissues (Figure 4D). Normal spinal tissue morphology was evident in control mice, but inflammatory infiltration was observed in EAE-induced mice treated with physiological saline (black arrows pointing to the pink regions in H&E staining). Mice treated with Dab or PAR-1-inh showed an absence of inflammatory infiltration, suggesting that the anti-coagulants attenuated inflammation in the spinal cord. In terms of LFB staining, vehicle-treated mice showed a much weaker intensity of blue staining, revealing that EAE induced remarkable demyelination of the spinal tissues. However, the intensity of LFB staining was recovered by Dab and PAR-1-inh, which implies that the anti-coagulants had a protective effect against demyelination in EAE.

### *In vivo* Effect of Dabigatran on EAE is Mediated by PAR-1/SphK/S1P Signaling Axis

**Figure 5:**
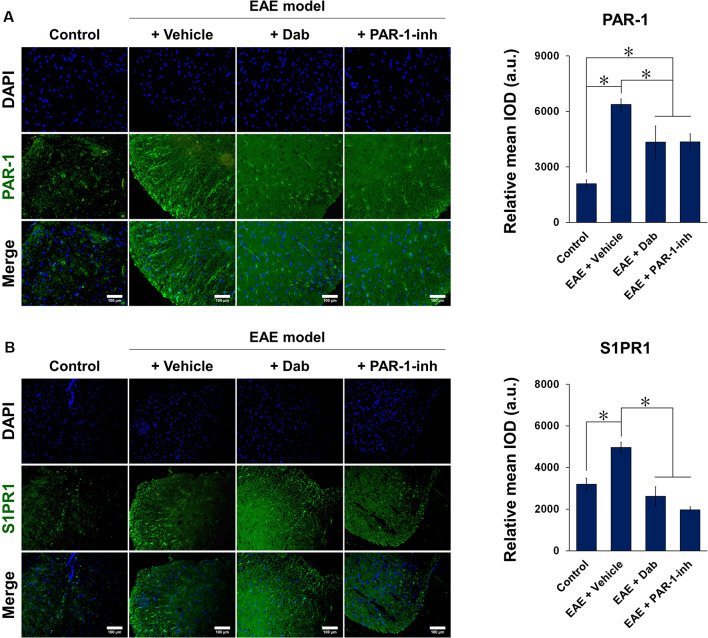
Immunofluorescence of PAR-1 and S1PR1 in spinal cord tissues of mice induced by EAE. Mice subjected to EAE modeling were treated with vehicle (physiological saline), Dab, or PAR-1-inh. Tissues were stained for **(A)** PAR-1 and **(B)** S1PR1 in green and nuclei (DAPI) in blue. Scale bar = 100 μm. Green fluorescence was quantified by measuring the relative mean IOD. The data are presented as the mean ± SD (*n* = 6), *p < 0.05.

**Figure 6:**
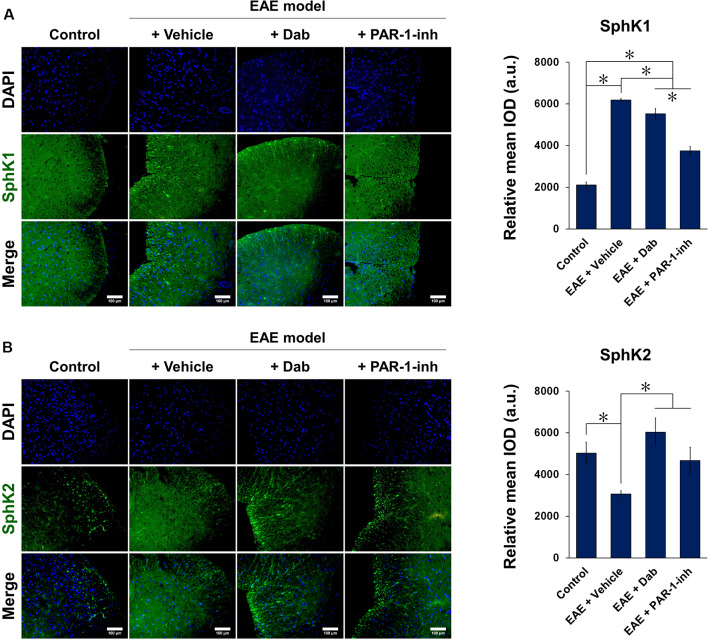
Immunofluorescence of SphK1 and SphK2 in spinal cord tissues of mice induced by EAE. Mice subjected to EAE modeling were treated with vehicle (physiological saline), Dab, or PAR-1-inh. Tissues were stained for **(A)** SphK1 and **(B)** SphK2 in green and nuclei (DAPI) in blue. Scale bar = 100 μm. Green fluorescence was quantified by measuring the relative mean IOD. The data are presented as the mean ± SD (*n* = 6), *p < 0.05.

Figures 5, 6 show the immunofluorescence staining of PAR-1 and S1P signaling-related proteins (S1PR1, SphK1, and SphK2) in spinal cord tissues extracted from mice subjected to EAE modeling. Compared with control animals, EAE induction (“+Vehicle” group) resulted in a marked increase in the expression of PAR-1, S1PR1, and SphK1 and a significant decrease in that of SphK2 in spinal cord tissues. Upon Dab or PAR-1-inh administration, the expression of PAR-1, S1PR1, and SphK1 was significantly downregulated compared to that in mice treated with a vehicle, whereas that of SphK2 was significantly upregulated. These results are in agreement with the *in vitro* data and revealed the involvement of the blood coagulation pathway (PAR-1) in the regulation of EAE through S1P signaling.

## Discussion

Coagulation pathways have been demonstrated to be associated with inflammation and autoimmunity, regulating hemostasis and contributing to the pathogenesis of MS (Ziliotto et al., 2019). In this study, LPS and thrombin were applied to induce the activation of astrocytes. LPS is a common inducer of neuroinflammation (Catorce and Gevorkian, 2016; Sheppard et al., 2019), and thrombin at low physiological levels exert neuroprotective effects while contributing to neurodegeneration at high doses (Striggow et al., 2000; Plantone et al., 2019; Gofrit and Shavit-Stein, 2019). Through our *in vitro* study, we suggest that the effect of LPS and thrombin may be mediated by the blood coagulation cascade (PAR-1) and can be counteracted by anti-coagulants, with Dab exerting similar effects as PAR-1 antagonization. Additionally, we elucidated the mechanism by which the coagulation cascade participates in immune function in the EAE model, namely through the association of PAR-1 with the S1P metabolic pathway. Also, Dab, a clinical oral anti-coagulant that acts as a reversible inhibitor of thrombin, was used for the first time to treat EAE-induced mice. Clinical trials involving the treatment of a variety of conditions and diseases, such as pulmonary embolism and chronic atrial fibrillation, have been carried out with Dab, demonstrating the therapeutic benefit of Dab (Redondo et al., 2011). The findings of our study further offer important translational prospects for the clinical treatment and management of MS.

S1P is a metabolic intermediate product of phospholipids, and its production is catalyzed by rate-limiting enzymes known as SphKs (subtypes SphK1 and SphK2; Karliner, 2006). In addition to acting as an extracellular lipid mediator (Chun and Hartung, 2010), S1P functions as an important secondary signaling molecule that induces physiological effects by binding with its receptors S1PR1–5. These receptors are members of the G protein-coupled receptor family (O’Sullivan and Dev, 2013) that activate different pathways in astrocytes and other cells (Bryan and Del Poeta, 2018; Grassi et al., 2019). S1PR1 has become a common target in MS research as ample evidence has demonstrated its role in regulating MS progression. For example, Gonzalez-Cabrera et al. (2012) showed that specific S1PR1 antagonism alleviated EAE, and S1PR1-mediated neuroinflammatory response has gained widespread focus (Garris et al., 2013; Liu et al., 2016). Of particular importance, the S1P-mimicking agent fingolimod (FTY720), an immunosuppressive drug against MS, is phosphorylated by SphK2 *in vivo* (Paugh et al., 2003). The phosphorylated form non-selectively activates S1PR1 on lymphocytes, which then causes the receptors to be internalized and their functions to be antagonized (O’Sullivan et al., 2018). This internalization in turn regulates lymphocytic egress and attenuates abnormal immune function in EAE (Garris et al., 2014). Additionally, the non-immunological role of FTY720 in regulating the CNS was reported by Choi et al. (2011) who showed that FTY720 alleviated the symptoms of EAE by inhibiting the binding between S1P and S1PR1 on astrocytes. This finding is critical as the levels of S1PRs in the CNS are higher than those in peripheral blood, and FTY720 can penetrate the blood-brain barrier to act directly on the CNS (Rothhammer et al., 2017). Taken together, the interaction between S1P and S1PR1 on astrocytes is considered to be the core link in astrocyte activation and EAE pathogenesis.

It is important to examine the activity of SphKs in astrocytes in understanding their participation in EAE pathogenesis. Studies have suggested that SphK1 and SphK2 play opposing roles in sphingolipid metabolism, and their effect on apoptosis has also been revealed, SphK1 being pro-survival and SphK2 being pro-apoptosis (Maceyka et al., 2005; Hait et al., 2006). In particular, SphK2 has shown potent anti-inflammatory effects by regulating macrophage activation and polarization (Ji et al., 2019; Weigert et al., 2019). Moreover, the cellular localization of the SphKs was shown to be associated with epithelial ovarian cancer (Xu, 2018), Sandhoff disease (Wu et al., 2008), and Alzheimer’s disease (Dominguez et al., 2018). Based on the fact that the anti-MS action of FTY720 is catalyzed by SphK2 as mentioned above (Fischer et al., 2011), it is reasonable to suggest that SphK2 has neuroprotective functions in MS and EAE. In our study, the expression and release of S1P in astrocytes and spinal cord tissue were inversely correlated to the expression of SphK2. Our findings thus indicate that aggravation of neuronal dysfunction in the EAE model is accompanied by decreased SphK2 expression, whereas its upregulation is associated with restoration of neuronal function.

Dab is an oral anti-coagulant drug that is included on the World Health Organization’s Model List of Essential Medicines. Approved in 2010, it is mainly applied in the prevention of stroke in individuals with atrial fibrillation (Hernandez et al., 2015). Given the strong link between coagulation, inflammation, and autoimmunity, we proposed that Dab, a direct inhibitor of thrombin (Bogatkevich et al., 2009), may exert therapeutic properties against EAE through coagulation-associated signaling. We showed that Dab downregulated the expression of PAR-1 in LPS- and thrombin-induced astrocytes and in EAE-induced mice, and the effect of Dab was similar to that of SCH 530348, a PAR-1 inhibitor. Because PAR-1 is a receptor of thrombin (Shea et al., 2017), the inhibition of thrombin by Dab effectively limited the activation of PAR-1. Importantly, the interplay between PAR-1 and S1P is critically implied in a variety of biological phenomena. For example, S1PR1 is required for PAR-1-mediated endothelial barrier stabilization, which is dependent on SphK1 (Feistritzer and Riewald, 2005). Also, thrombin led to the transient activation of SphK1 and S1P in lung epithelial cells through PAR-1 signaling (Billich et al., 2009).

Studies on the role of the PAR-1/S1P signaling axis in astrocytes and EAE remain scarce. Herein, we investigated the anti-EAE effect of Dab in primary astrocytes and EAE-induced mice. *In vitro*, we found that Dab had an anti-inflammatory effect as it reduced the expression of IL-1β in astrocytes. While LPS and thrombin triggered the activation of astrocytes, Dab suppressed this activation by decreasing the expression of PAR-1, subsequently leading to the downregulation of S1PR1 and SphK1 and the recovery of SphK2 expression over time. Similar phenomena were observed *in vivo*, where the effect of Dab was analogous to that of PAR-1-inh in terms of recovering neurological function, suppressing inflammatory reaction, and preventing spinal demyelination in EAE-induced mice through S1P-associated signaling.

Despite the comprehensive data presented here, our study suffers from several limitations. The first limitation is the lack of data on the cellular and subcellular localization of the SphKs, which may provide valuable information on the mechanism of the PAR-1/SphK/S1P axis. Second, while the effect of Dab on SphK1 expression has been studied in the literature, the interaction between Dab and SphK2 remains elusive and requires in-depth evaluation. Third, a systematic study of other S1P receptors will provide a broader look at the mechanism of the PAR-1/SphK/S1P axis in MS and EAE. These questions will form the basis of prospective follow-up research.

In summary, we showed that anti-coagulant drugs, in particular dabigatran, ameliorated CNS inflammation and alleviated the symptoms of EAE and MS *via* PAR-1/SphK/S1P signaling. By gaining an in-depth understanding of the correlation between coagulation mechanisms and CNS immune diseases, we offer a novel therapeutic approach that is potentially translational in developing clinical treatment schemes against MS.

## Data Availability Statement

The datasets generated for this study are available on request to the corresponding author.

## Ethics Statement

The animal study was reviewed and approved by the Model Animal Research Institute at Wuhan Myhalic Biotechnology Co., Ltd.

## Author Contributions

RC and JL designed the study and analyzed all of the data. RC performed the experiments. XC, WL, and HY assisted in the *in vitro* experiments (cell culture, qRT-PCR, immunofluorescence, western blot, and ELISA). XL and JY were involved in the *in vivo* experiments. RC prepared the manuscript. JL revised the manuscript. All authors have read and approved the final version of this manuscript.

## Conflict of Interest

The authors declare that the research was conducted in the absence of any commercial or financial relationships that could be construed as a potential conflict of interest.
