# Supplementary material for: Dabigatran Suppresses PAR-1/SphK/S1P Activation of Astrocytes in Experimental Autoimmune Encephalomyelitis Model
Source: Front Mol Neurosci. 2020 Jun 30;13:114. doi: 10.3389/fnmol.2020.00114 (PMC7338760; doi:10.3389/fnmol.2020.00114)
Supplement: Supplementary file 1 [file Data_Sheet_1.DOCX]

Supplementary Material

# Supplementary Figures


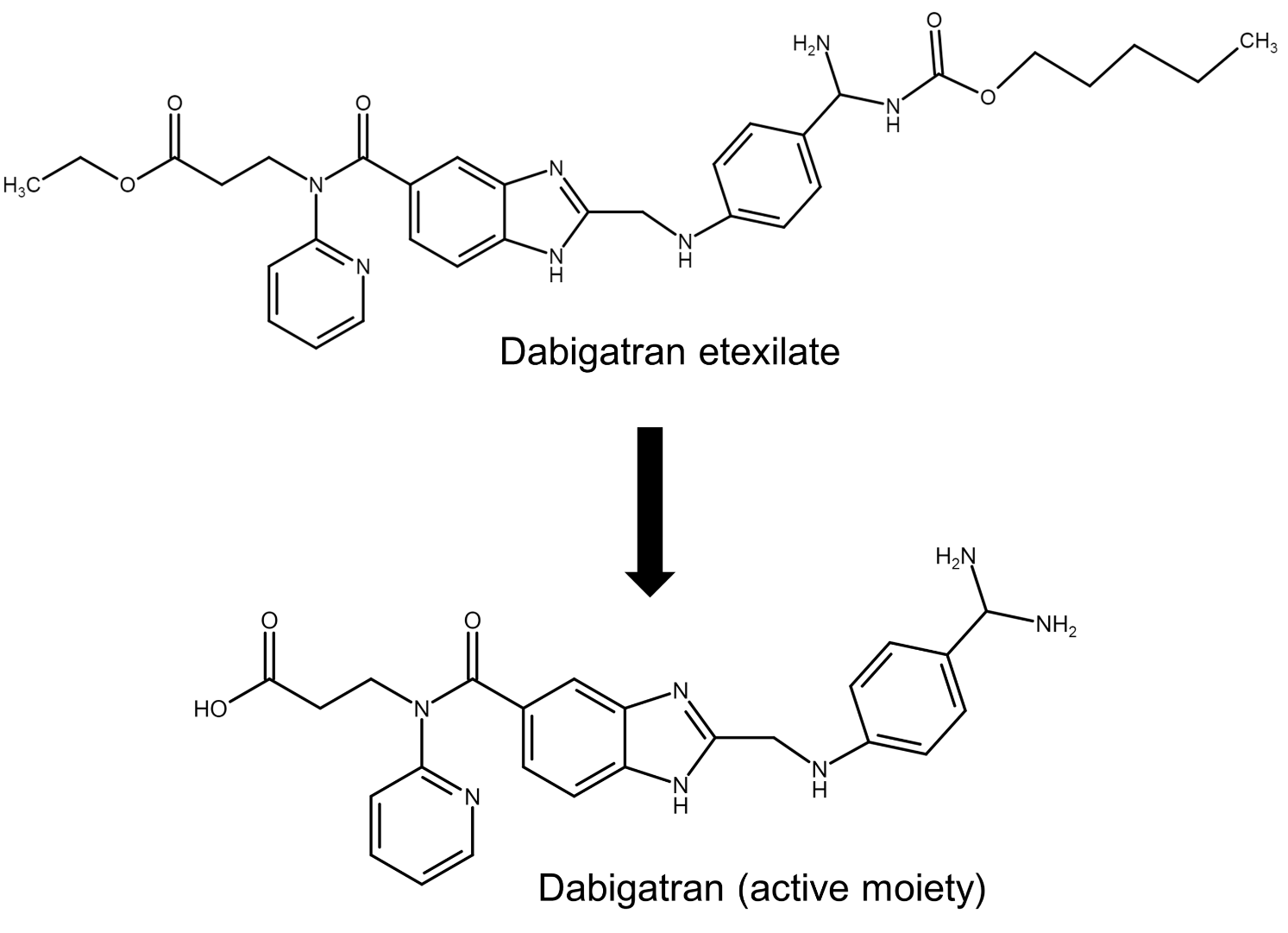


**Supplementary Figure S1.** Chemical structure of dabigatran etexilate and dabigatran. Dabigatran etexilate is the prodrug form that is administered orally and is hydrolyzed to active dabigatran moiety, which is a direct inhibitor of thrombin.

**
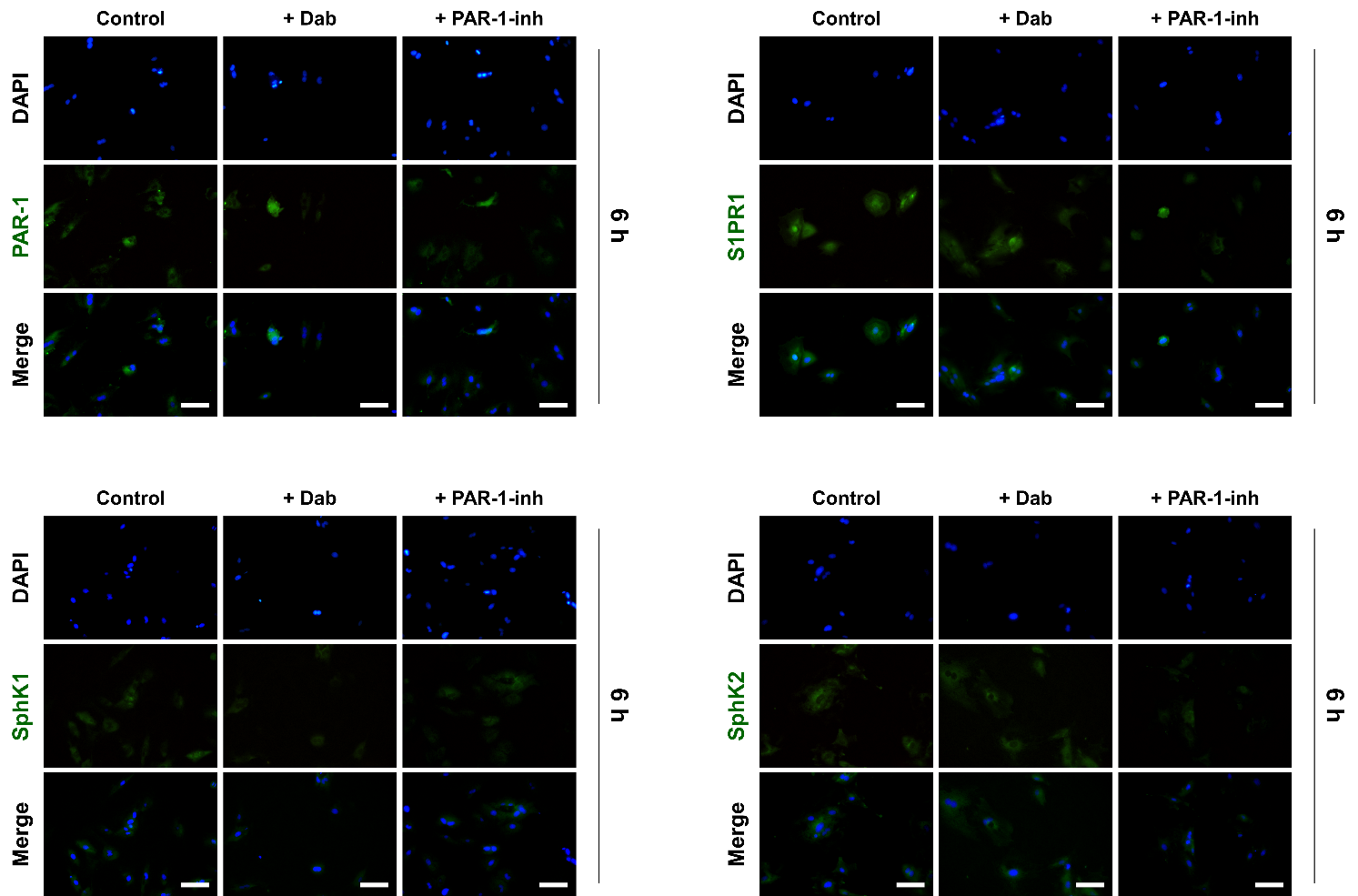
**

**Supplementary Figure S2.** Immunofluorescence of PAR-1, S1PR1, SphK1, and SphK2 in astrocytes. Astrocytes were treated with Dab or PAR-1-inh for 6 h. Cells were stained for PAR-1, S1PR1, SphK1, and SphK2 in green and nuclei (DAPI) in blue. Scale bar = 100 μm.


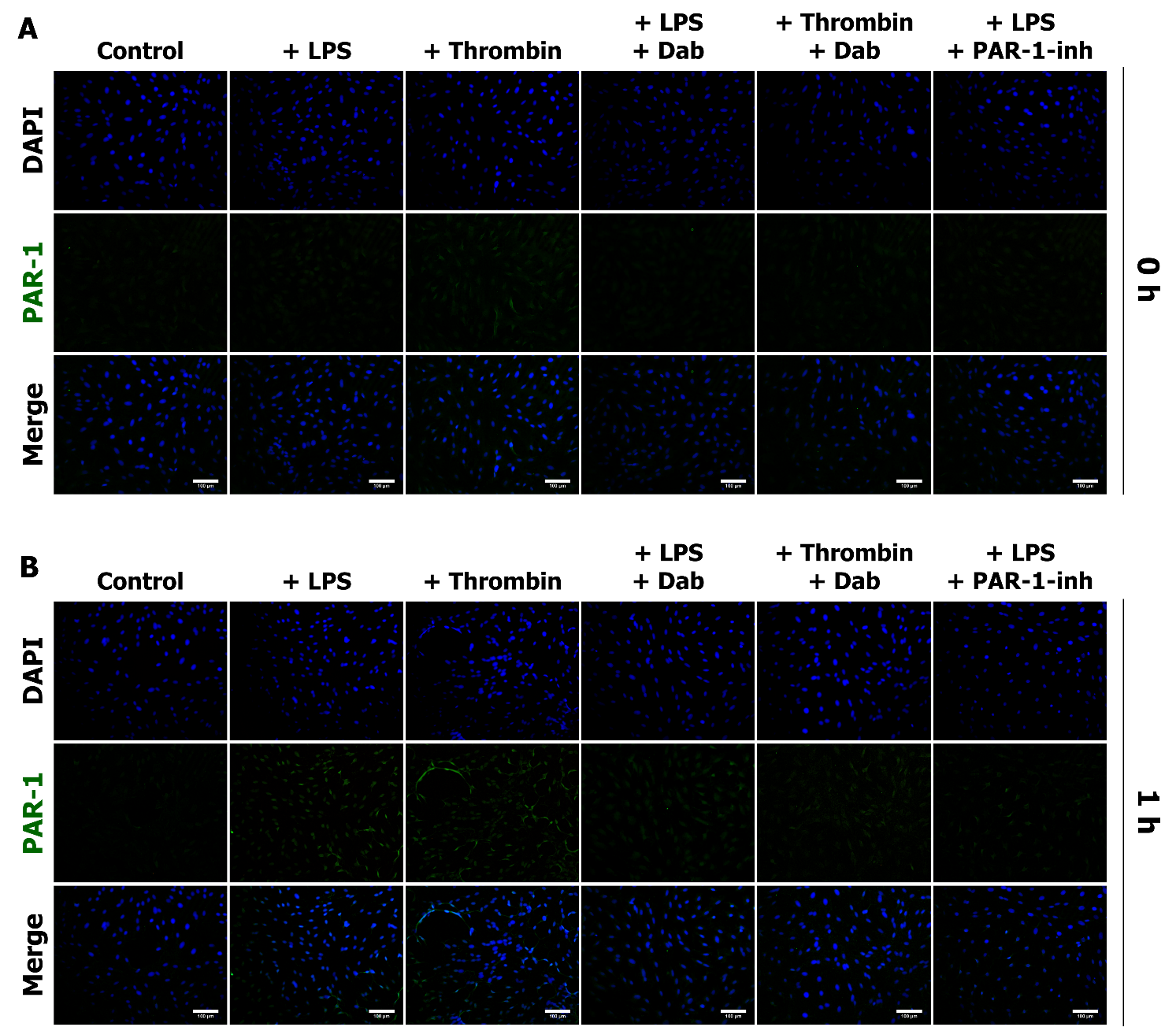


**Supplementary Figure S3.** Immunofluorescence of PAR-1 in astrocytes. Astrocytes were treated with LPS or thrombin, with or without Dab or PAR-1-inh (LPS only) for (A) 0 h or (B) 1 h. Cells were stained for PAR-1 in green and nuclei (DAPI) in blue. Scale bar = 100 μm.


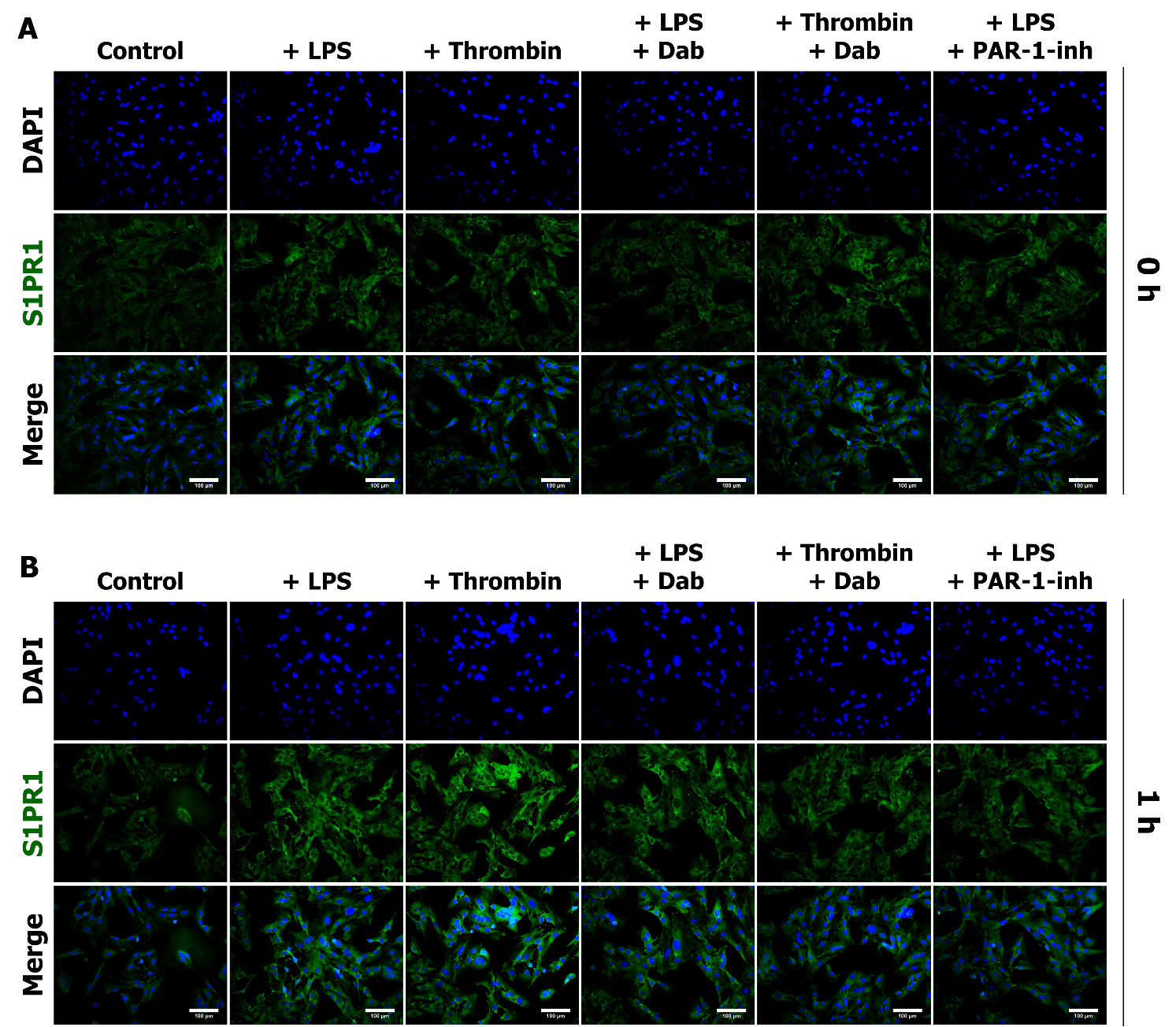


**Supplementary Figure S4.** Immunofluorescence of S1PR1 in astrocytes. Astrocytes were treated with LPS or thrombin, with or without Dab or PAR-1-inh (LPS only) for (A) 0 h or (B) 1 h. Cells were stained for S1PR1 in green and nuclei (DAPI) in blue. Scale bar = 100 μm.


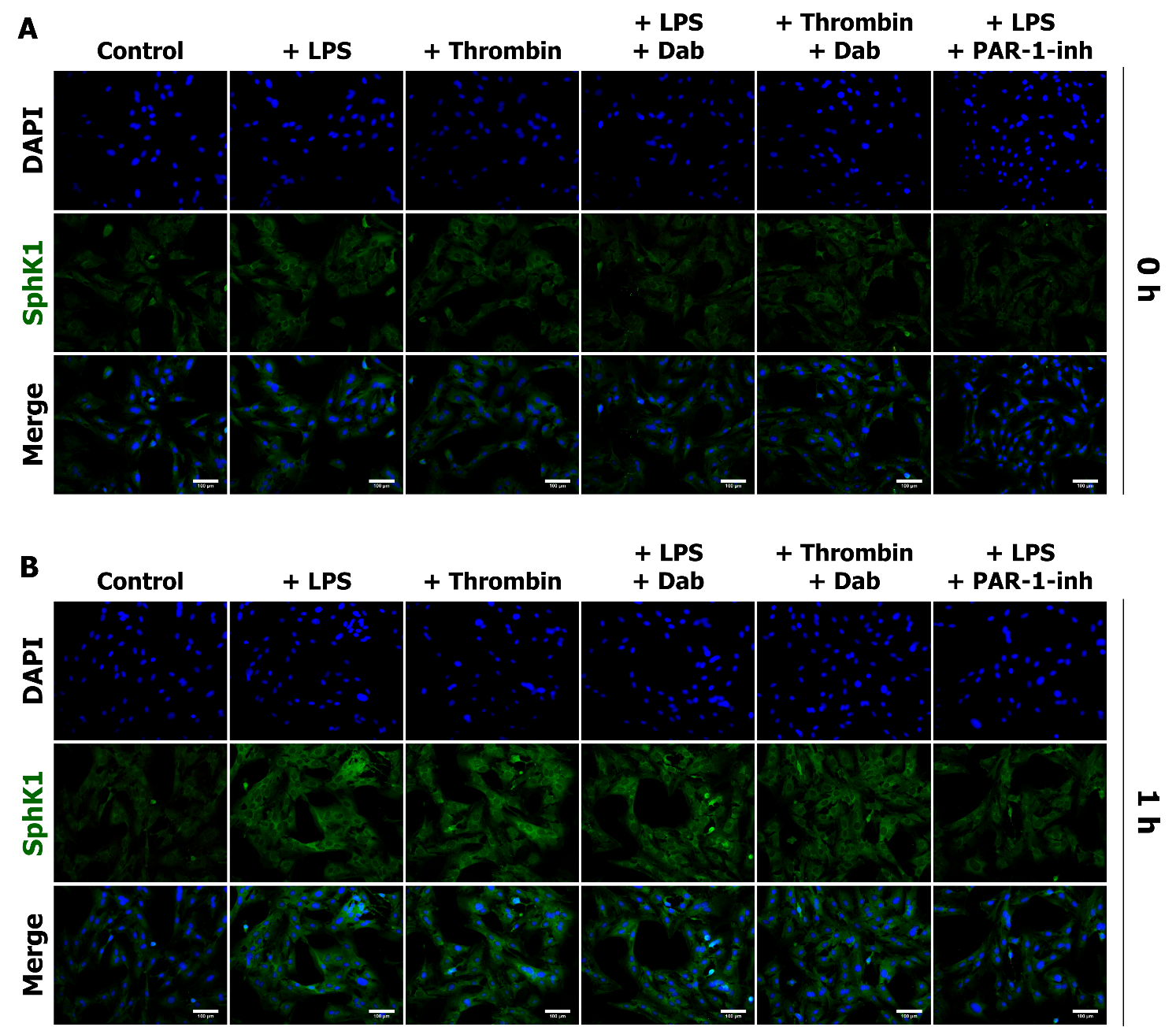


**Supplementary Figure S5.** Immunofluorescence of SphK1 in astrocytes. Astrocytes were treated with LPS or thrombin, with or without Dab or PAR-1-inh (LPS only) for (A) 0 h or (B) 1 h. Cells were stained for SphK1 in green and nuclei (DAPI) in blue. Scale bar = 100 μm.


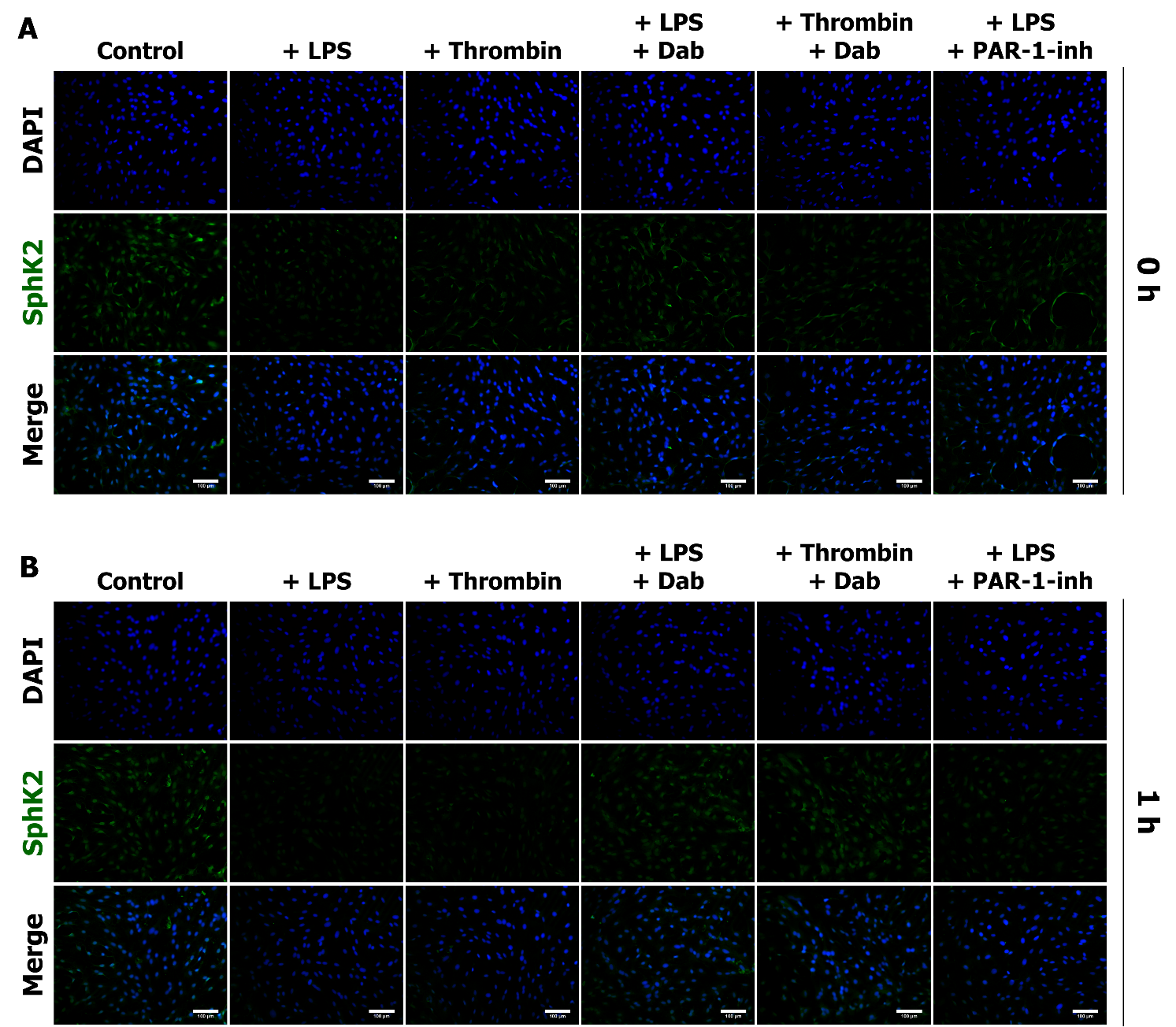


**Supplementary Figure S6.** Immunofluorescence of SphK2 in astrocytes. Astrocytes were treated with LPS or thrombin, with or without Dab or PAR-1-inh (LPS only) for (A) 0 h or (B) 1 h. Cells were stained for SphK2 in green and nuclei (DAPI) in blue. Scale bar = 100 μm.
